# ZEB2 facilitates peritoneal metastasis by regulating the invasiveness and tumorigenesis of cancer stem-like cells in high-grade serous ovarian cancers

**DOI:** 10.1038/s41388-021-01913-3

**Published:** 2021-07-01

**Authors:** Yiying Li, He Fei, Qiwang Lin, Fan Liang, Yanan You, Ming Li, Mengyao Wu, Ying Qu, Pengfei Li, Yan Yuan, Tong Chen, Hua Jiang

**Affiliations:** 1 Department of Gynecology, Obstetrics & Gynecology Hospital, Fudan University, Shanghai, 200011 China; 2 Department of Gynecology, The First Affiliated Hospital, Nanchang University, Nanchang, 330006 China; 3 Department of Gynecology, Shanghai Fifth People’s Hospital, Fudan University, Shanghai, 200240 China; 4 Department of Hematology, Huashan Hospital, Fudan University, Shanghai, 200040 China; 5 Shanghai Key Laboratory of Female Reproductive Endocrine Related Diseases, Shanghai, 200011 China

**Keywords:** Cancer stem cells, Ovarian cancer

## Abstract

Peritoneal metastasis is a common issue in the progression of high-grade serous ovarian cancers (HGSOCs), yet the underlying mechanism remains unconfirmed. We demonstrated that ZEB2, the transcription factor of epithelial–mesenchymal transition (EMT), was upregulated in ascites cells from HGSOC patients and in CD133^+^ cancer stem-like cells (CSLCs) from epithelial ovarian cancer (EOC) cell lines. SiRNA-mediated knockdown of ZEB2 in EOC cells decreased the percentage of CSLCs and reduced the colony forming potential, cell invasion capacity and expression of pluripotent genes Oct4 and Nanog. Inhibition of ZEB2 also induced cellular apoptosis and impacted the tumorigenicity of ovarian CSLCs. The mesenchymal markers N-cadherin and vimentin were downregulated, while the epithelial marker E-cadherin was upregulated after ZEB2 knockdown. MiR-200a, a molecule that downregulates ZEB2, had the opposite effect of ZEB2 expression in EOC-CSLCs. A retrospective study of 98 HGSOC patients on the relationship of ascites volume, pelvic and abdominal metastasis, International Federation of Gynecology and Obstetrics (FIGO) stage and the malignant involvement of abdominal organs and lymph nodes was performed. Patients with high expression of ZEB2 in tumour tissues had a higher metastasis rate and a poorer prognosis than those with low expression. The parameters of ZEB2 expression and ascites volume were strongly linked with the prognostic outcome of HGSOC patients and had higher hazard ratios. These findings illustrated that ZEB2 facilitates the invasive metastasis of EOC-CSLCs and can predict peritoneal metastasis and a poor prognosis in HGSOC patients.

## Introduction

Epithelial ovarian cancer (EOC) is the most lethal gynaecological malignancy [1]; high-grade serous tumours are a common subtype with aggressive characteristics [2]. Up to 70% of high-grade serous ovarian cancer (HGSOC) patients are in advanced stages with abdominal metastasis at diagnosis [3], and the 5-year survival rate is less than 30% [4]. Ascites is a clinical symptom highly related to the peritoneal spread of EOC cells [5]; the local microenvironment supports the engraftment and proliferation of these cells in distant regions [6]. Previous studies have focused on the composition of cellular and acellular factors in ascites, including immune evasion-related factors [7] and pro-inflammatory cytokines [8, 9] and chemokines [10], to reveal the mechanisms of abdominal metastasis in EOCs. However, as the underlying mechanism is highly complex and diverse, further exploration relating to the prognostic molecular biomarkers of HGSOC metastasis is of great interest.

Epithelial–mesenchymal (E–M) transition (EMT), a cellular remodelling programme in embryogenesis and tumorigenesis, contributes to tumour metastasis [11, 12], therapy resistance and disease recurrence [13, 14], during which cells lose epithelial apical–basal polarity and establish mesenchymal front–back polarity, decrease cell–cell adhesion and remodel cell–matrix adhesions as well as the cytoskeleton to acquire cell motility and invade the basement membrane [15, 16]. When tumour cells are shed from the primary lesion into the peritoneal cavity, EMT, as an essential process orchestrated by a series of transcripts, induces cellular anoikis resistance and leads to the survival of malignant cells in ascites [17].

The assessment of EMT should rely on the combination of the cell surface and multiple molecular markers, including Snai (Snail and Snai2), Zeb (Zeb1 and Zeb2) and Twist1 [16]. A shift from epithelial markers to mesenchymal features or hybrid E–M status represents E–M plasticity and helps to distinguish the transforming process. Among these markers, ZEB2, the Zn-finger EMT transcription factor, is highly expressed in metastatic ovarian cancer cells compared to in situ lesion cells, indicating that ZEB2 plays an important role in ovarian cancer peritoneal metastasis [18–20]. Furthermore, a class of small non-coding RNAs, the microRNA (miR)-200 family, has been identified as a family of post-transcriptional inhibitors of ZEB2. The reciprocal feedback loop between ZEB2 and miR-200s is tightly implicated in the EMT and invasive potential of malignancies [21]. However, the mechanism by which miR-200/ZEB2 affects HGSOC progression remains largely unknown.

In this study, we investigated the effects of miR-200a/ZEB2 on the invasion of ovarian cancer stem-like cells (CSLCs) by regulating the expression of ZEB2 in ovarian cancer cell lines. The prognostic value of ZEB2 expression in 98 HGSOC patients was estimated by a retrospective study. Our results suggested that ZEB2 participates in peritoneal metastasis by regulating the invasive capacity of CSLCs and serves as an effective biomarker for HGSOC prognosis prediction.

## Results

### ZEB2 and CSLCs played a facilitative role in the peritoneal metastasis of HGSOC

**Fig. 1:**
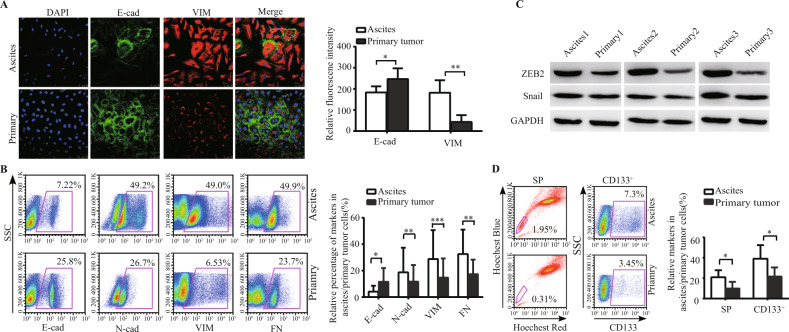
ZEB2 and CSLCs played a facilitative role in the peritoneal metastasis of HGSOC. **A** Immunofluorescence staining of E-cad (green) and VIM (red) in HGSOC primary tumour cells and ascitic cells (left panel). The median fluorescence intensity was analysed by ImageJ (*N* = 6 in each group) (right panel). **B** FACS analysis of E-cad, N-cad, VIM and FN in paired ascites and primary tumour cell samples from six HGSOC patients. Representative FACS figures are shown (left panel), and the grouped relative percentages of markers were analysed (right panel). **C** Expression of ZEB2 and Snail in paired ascites and primary tumour cell samples from three HGSOC patients was analysed by western blotting. **D** FACS analysis of SP and CD133^+^ subpopulations in paired ascites and primary tumour cell samples from six HGSOC patients. Representative images are shown (left panel), and the grouped relative percentages of markers were analysed (right panel). The data represented the mean ± SD. The experiments were repeated independently at least three times. *p < 0.05, **p < 0.01, ***p < 0.001.

We first collected HGSOC cells from ascitic fluid and primary tumours. Expression of the ovarian cancer markers carbohydrate antigen 125 (CA125) and human epididymis protein 4 (HE4) was determined by reverse transcription-polymerase chain reaction (RT-PCR) and western blot analyses. Similar to a previous study [22, 23], cells from ascites expressed either CA125 or HE4, while human skin fibroblasts (HSFs) did not express these markers (Fig. S1A, B), indicating that the cells collected following our protocol were indeed ovarian cancer cells. To analyse the EMT status between the primary and peritoneal metastatic lesions, the expression of the epithelial marker E-cadherin (E-cad) and the mesenchymal markers N-cadherin (N-cad), vimentin (VIM) and fibronectin (FN) was investigated in tumour cells derived from ascitic fluid and primary tumours. Ascitic cells displayed a more elongated and polygonal shape than primary HGSOC tumour cells, showing lower expression of E-cad (green) and higher expression of VIM (red) (Fig. 1A). According to analyses of fluorescence-activated cell sorter (FACS) and RT-PCR, ascitic cells expressed higher levels of mesenchymal markers including N-cad, VIM and FN (*p* < 0.01, <0.001, <0.01, respectively), while cells from the primary tumour expressed higher levels of epithelial marker E-cad (*p* < 0.05) (Figs. 1B, S1C). EMT-related transcription factors were additionally compared between ascitic cells and cells from primary tumours. ZEB2 was elevated in ascitic cells, while the changes in ZEB1, Snail and Twist1 in ascites and primary tumours were mild (Fig. 1C, S1D). In addition, the expression of invasive factors matrix metalloproteinase-2 and 9 (MMP2, MMP9) was increased in ascitic cells compared to cells from primary tumours (Fig. S1E, F).

Several markers and assays are being applied to assess tissue stem cells. Among them, a functional Hoechst 33342 dye-effluxing assay [24] and the cell surface marker CD133 [25] have been well established for purifying EOC-CSLCs. In our previous study, ZEB2 was significantly increased in Hoechst 33342-effluxing side population (SP) cells compared to non-SP cells, and the percentage of SP cells was higher in ascites than that in primary tumours [26]. In this study, either CD133^+^ cells or SP cells were designated CSLCs. Both SP cells and CD133^+^ cells were present in higher proportions in the ascites than in their paired primary tumours from HGSOC patients (*p* < 0.05, Fig. 1D). These findings indicated that ZEB2 and CSLCs are elemental factors in HGSOC peritoneal metastasis.

### ZEB2 was upregulated in EOC-CSLCs and correlated with their abundance, mesenchymal features and invasive potential

**Fig. 2:**
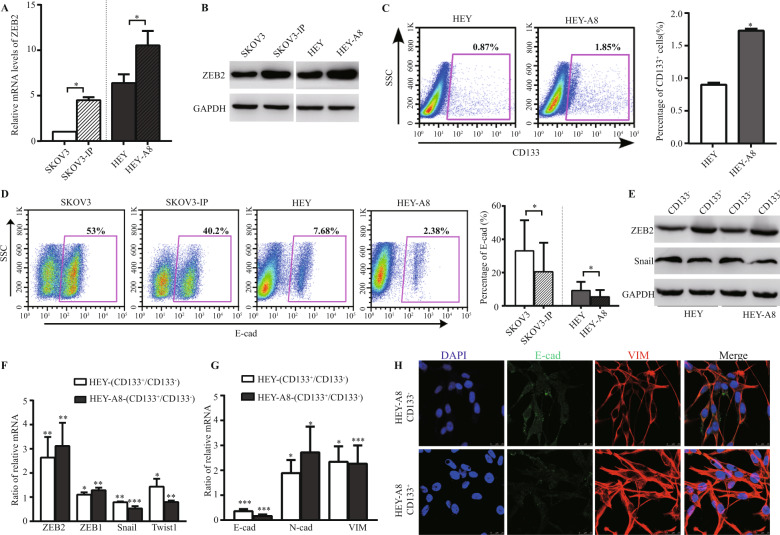
ZEB2 was upregulated in EOC-CSLCs and correlated with their abundance, mesenchymal features and invasive potential. **A** The expression of ZEB2 in the SKOV3 and HEY cell lines and their daughter lines, SKOV3-IP and HEY-A8, was analysed by RT-PCR and normalised to the levels measured in the SKOV3 cell line (=1). **B** Expression of ZEB2 in SKOV3, SKOV3-IP, HEY and HEY-A8 cells were analysed by western blotting. **C** The CD133^+^ subpopulation in HEY and HEY-A8 cell lines were analysed by FACS. Representative FACS figures are shown (left panel), and the grouped percentages of CD133^+^ cells were analysed (right panel). **D** Dot plot (left panel) and grouped percentages of E-cad expression (right panel) in SKOV3, SKOV3-IP, HEY and HEY-A8 cells were analysed by flow cytometry analysis. **E** Expression of ZEB2 and Snail in CD133^+^ and CD133^−^ cells of HEY and HEY-A8 cell lines were analysed by western blotting. **F** The relative mRNA levels of ZEB1, ZEB2, Snail and Twist1 in CSLCs relative to paired NCSLCs (CD133^+^/CD133^−^) in HEY and HEY-A8 cell lines were analysed by RT-PCR. **G** The relative mRNA levels of E-cad, N-cad and VIM in CSLCs relative to paired NCSLCs (CD133^+^/CD133^−^) were analysed by RT-PCR. **H** Immunofluorescence staining of E-cad (green), VIM (red) and DAPI (blue) in CD133^+^ and CD133^−^ HEY-A8 cell lines. Scale bar = 25 μm. The data represented the mean ± SD. The experiments were repeated independently at least three times. *p < 0.05, **p < 0.01, ***p < 0.001.

To explore the potential mechanisms of ZEB2 in EOC-CSLC-initiated peritoneal metastasis, we analysed ZEB2 expression in human ovarian cancer cell lines and their metastatic daughter lines (SKOV3/SKOV3-IP and HEY/HEY-A8). We found that all metastatic daughter cells, SKOV3-IP and HEY-A8, had higher ZEB2 expression (Fig. 2A, B) and an increased percentage of CD133^+^ cells (HEY vs HEY-A8: 0.9 ± 0.03 vs 1.73 ± 0.03, *p* < 0.05, Fig. 2C), whereas the mother lines exhibited a more epithelial phenotype showing higher E-cad expression and lower N-cad, VIM, MMP2 and MMP9 expression (Fig. 2D, S2A–E). CD133^+^ CSLCs had higher ZEB2 levels than CD133^−^ non-CSLCs (NCSLCs) (Fig. 2E, F). HEY-CD133^+^ and HEY-A8-CD133^+^ cells exhibited more mesenchymal features with N-cad and VIM expression (Fig. 2G, H). However, the elevated expression of ZEB2 in CD133^+^ cells indicated that ZEB2 may play an important role in regulating ovarian CSLC-driven pathogenesis.

### ZEB2 enhanced the tumorigenesis and metastatic potential of EOC-CSLCs

We then efficiently knocked down ZEB2 by transfecting siRNA-ZEB2 (shZEB2) lentiviral vectors in the SKOV3-IP and HEY-A8 lines to explore the impact of ZEB2 on ovarian CSLC function (Fig. S3A, B). The E–M markers and pluripotency of CSLCs were determined after ZEB2 inhibition.

**Fig. 3:**
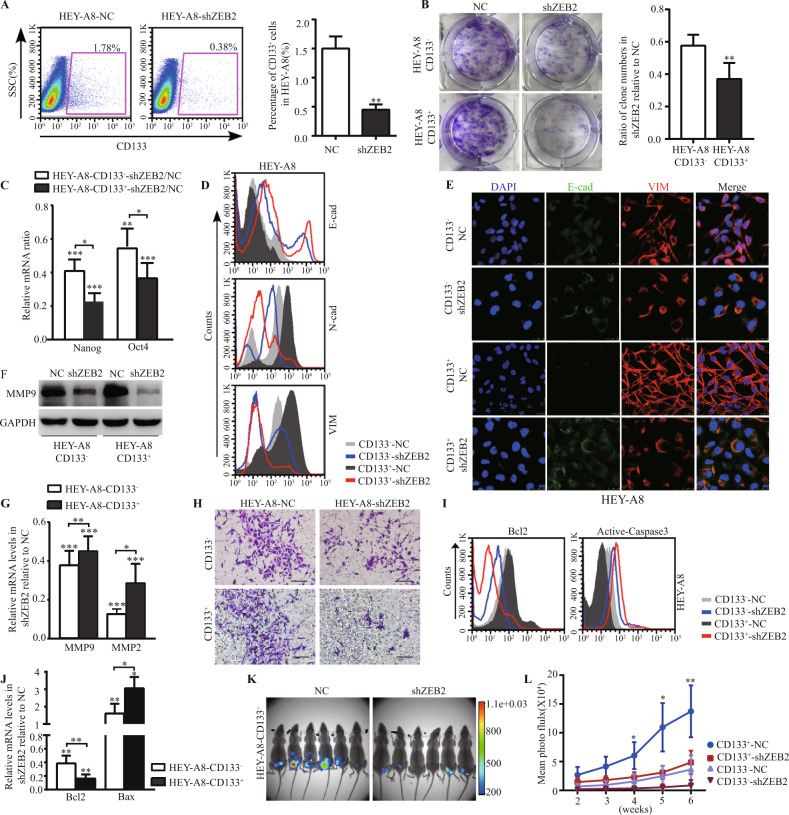
ZEB2 enhanced the tumorigenesis and metastatic capacity of EOC-CSLCs. **A** FACS analysis of CD133^+^ subpopulations in shZEB2- or NC-transfected HEY-A8 cells. Representative FACS figures are shown (left panel), and the relative grouped percentages were analysed (right panel). **B** Colony formation assay of CD133^+^ and CD133^−^ cells in shZEB2- or NC-transfected HEY-A8 cells (left panel). The percentage of colony number in shZEB2- and NC-transfected HEY-A8 cells were compared (right panel). **C** The relative mRNA levels of Nanog and Oct4 in CD133^+^/CD133^−^ cells from shZEB2-transfected HEY-A8 cells was compared to their NC counterparts. **D** FACS analysis of E-cad, N-cad and VIM in CD133^+^/CD133^−^ cells purified from shZEB2- or NC-transfected HEY-A8 cells. **E** Immunofluorescence staining of E-cad (green), VIM (red) and DAPI (blue) in CD133^+^ or CD133^−^ cells purified from shZEB2/NC-transfected HEY-A8 cells. Scale bar = 25 μm. **F** Expression of MMP9 in CD133^+^ or CD133^−^ cells from the shZEB2-HEY-A8 line was analysed by western blotting. **G** The relative mRNA of MMP9 and MMP2 in CD133^+^/CD133^−^ cells from shZEB2-transfected HEY-A8 cells was compared to their NC counterparts. **H** Transwell assay of CD133^+^/CD133^−^ cells purified from shZEB2/NC-transfected HEY-A8 cells. Scale bar = 100 μm. **I** FACS analysis of Bcl2 and active caspase-3 in CD133^+^ or CD133^−^ cells from the shZEB2-HEY-A8 line. **J** The relative mRNA of Bcl2 and Bax in CD133^+^/CD133^−^ cells from shZEB2-transfected HEY-A8 cells was compared to their NC counterparts. **K** Bioluminescence image of xenografted mice 6 weeks after coeliac injection with CD133^+^ cells from the shZEB2/NC-transfected HEY-A8 line. The bar with the colour gradient indicates the fluorescence intensity. **L** The fluorescent intensity of engrafted tumours was calculated weekly by the luciferase in vivo imaging technique and is shown in the growth curve (*n* = 6 for each group). The data represent the mean ± SD. Each experiment was independently repeated at least three times. *p < 0.05, **p < 0.01, ***p < 0.001.

In shZEB2-transfected sublines, the percentages of CD133^+^ cells were significantly decreased compared with the non-specific control (NC-HEY-A8 vs shZEB2-HEY-A8: 1.5 ± 0.21 vs 0.45 ± 0.09, *p* < 0.01, Fig. 3A). The colony forming capacity and expression of the pluripotent genes Nanog and Oct4 were significantly reduced in CSLCs but not in NCSLCs (Fig. 3B, C, S3C). N-cad and VIM were consequently downregulated (Fig. S3D). Specifically, the trend of increasing E-cad and decreasing N-cad and VIM was much more prominent in CSLCs than in NCLSCs after ZEB2 was inhibited (Fig. 3D, E). These findings demonstrated that ZEB2 exerts important functions to help ovarian CSLCs sustain their pluripotency and E–M properties.

It has been indicated that peritoneal metastatic potential is related not only to the pluripotency but also to the mobility and anti-apoptotic capacity of CSLCs. We found that in shZEB2-transfected cell lines, the invasive capacity of CSLCs dramatically decreased compared to that of NCSLCs, in accordance with the downregulated expression of MMP9 and MMP2 in shZEB2-transfected CD133^+^ cells (Fig. 3F–H). To determine the cellular anti-apoptotic capability, serum-free media were applied to the culture system at 4 °C for 24 h. Analyses of Bcl2, Bax and annexin V/7-AAD apoptosis assays were performed. Bcl2 was decreased, while active caspase-3 was elevated in shZEB2-transfected CSLCs (Fig. 3I, J). Likewise, the changes in annexin V^+^ cells among shZEB2-transfected CD133^+^ cells were more conspicuous than those among shZEB2-transfected CD133^−^ cells (Fig. S3E).

To further investigate the function of ZEB2 in peritoneal metastasis, ovarian CD133^+^ cells derived from luciferase-labelled shZEB2- and negative control (NC)-transfected HEY-A8 cells were intraperitoneally injected into NOD/SCID mice. Overall, 4 of 6 mice injected with shZEB2-transfected CD133^+^ cells had fewer luciferase-labelled tumours and weaker signals, while 6/6 mice injected with the same numbers of NC-CD133^+^ cells showed massive clusters of luciferase signals (Fig. 3K). These mice were monitored for 6 weeks. The mean fluorescence intensity of the mice injected with NC-CD133^+^ cells was four times that of shZEB2-transfected CD133^+^ mice at week 3 and increased to 13.7-fold at week 6, while it was only 1.7- and 4.9-fold in the NC-CD133^−^ and shZEB2-transfected CD133^−^ groups, respectively (Fig. 3L), indicating that inhibition of ZEB2 represses EOC metastatic capacity.

### MiR-200a impacted the pluripotency and invasiveness of EOC-CSLCs

**Fig. 4:**
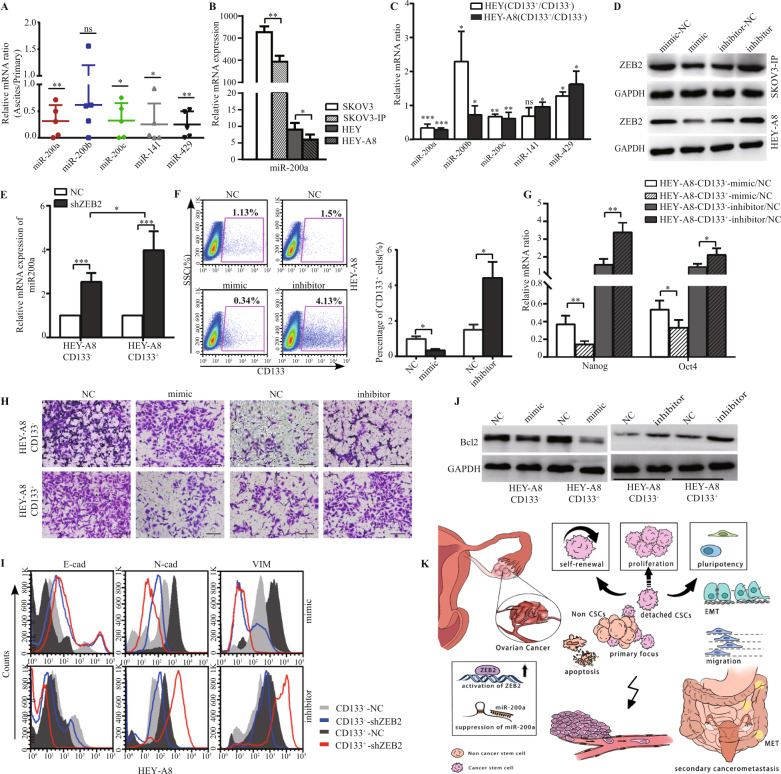
MiR-200a impacted the pluripotency and invasiveness of EOC-CSLCs. **A** The relative mRNA levels of miR-200 family members in ascites cells relative to paired primary tumour cells derived from HGSOC patients were analysed by RT-PCR. **B** Expression of miR-200a in SKOV3, HEY and their daughter lines SKOV3-IP, HEY-A8 was analysed by RT-PCR. **C** The relative mRNA levels of miR-200 family members in CSLCs relative to paired NCSLCs in HEY and HEY-A8 cell lines were analysed by RT-PCR. **D** The expression of ZEB2 in miR-200a-mimic/inhibitor-transfected SKOV3-IP and HEY-A8 cells was analysed by western blotting. **E** The relative mRNA of miR-200a in CD133^+^/CD133^−^ cells purified from shZEB2-transfected HEY-A8 cells was analysed by RT-PCR and normalised to their NC-transfected counterparts (=1). **F** FACS analysis of CD133^+^ subpopulations in miR-200a-mimic/inhibitor-transfected HEY-A8 cells. Representative FACS figures are shown (left panel), and the grouped percentages of CD133^+^ cells were analysed (right panel). **G** Expression of Nanog and Oct4 in miR-200a-mimic/inhibitor-transfected CD133^+^/CD133^−^ cells from HEY-A8 cells. **H** Transwell assay of CD133^+^ or CD133^−^ cells derived from the miR-200a-mimic/inhibitor-transfected HEY-A8 line. Scale bar = 100 μm. **I** FACS analysis of E-cad, N-cad and VIM in CD133^+^ or CD133^−^ cells derived from the miR-200a-mimic/inhibitor-transfected HEY-A8 line. **J** Western blot analysis of Bcl2 in CD133^+^/CD133^−^ cells derived from miR-200a-mimic/inhibitor-transfected HEY-A8 lines. **K** Schematic diagram of the regulatory activity of miR-200a/ZEB2 in CSLC-initiated peritoneal metastasis. The data represented the mean ± SD. Each experiment was repeated independently at least three times. *p < 0.05, **p < 0.01, ***p < 0.001.

MiR-200s are inhibitory miRNAs targeting ZEB2. There are five members of the miR-200 family, including miR-200a, miR-200b, miR-200c, miR-429 and miR-141, that compose a feedback loop and maintain the epithelial cell state [27]. We further investigated whether miR-200s exert suppressive functions on the tumorigenesis and metastasis of EOCs. Interestingly, compared to other miR-200 family members, miR-200a was expressed at lower levels in ascitic cells and highly metastatic daughter cells (Fig. 4A, B), and was significantly reduced in CD133^+^ cells (Fig. 4C). We thus focused on miR-200a and dissected the interaction between miR-200a and ZEB2.

First, mimics (10 nM, Qiagen, Germany) and inhibitors (50 nM, Qiagen, Germany) of miR-200a were transfected into HEY-A8 cells (Fig. S4A). MiR-200a mimics downregulated ZEB2, while miR-200a inhibitors upregulated ZEB2 expression (Fig. 4D), and both of the changes were more significant in CD133^+^ cells than in CD133^−^ cells (Fig. S4B). In addition, in shZEB2-transfected CD133^+^ cells, miR-200a was higher than that in CD133^−^ NCSLCs (Fig. 4E), indicating that the inverse coupling relationship between miR-200a and ZEB2 is more linked in CSLCs.

Second, the pluripotency of CSLCs was assessed after they were transfected with miR-200a mimics and inhibitors. The percentages of CD133^+^ cells were decreased by miR-200a (Fig. 4F). The inhibitory effect with respect to pluripotent gene expression was more significant in CD133^+^ CSLCs than in CD133^−^ NCSLCs (Fig. 4G). We further investigated the impact of miR-200a on the invasiveness of CD133^+^ cells, another factor regulating malignant cell tumorigenic and metastatic ability. The miR-200a mimics decreased the invasive capacity of CD133^+^ cells (Fig. 4H) and enhanced epithelial E-cad expression but not N-cad and Vim expression, and these results were the opposite of those under conditions with miR-200a inhibitors (Fig. 4I).

Third, Bcl2-related cellular apoptosis was analysed after mimic/inhibitor transfection. In the cells transfected with miR-200a-mimic or inhibitor, Bcl2 was reduced or elevated, respectively (Figs. 4J, S4C, D). All these data provide evidence that miR-200a exerts an impeditive effect on EOC-CSLC-originated tumorigenesis by inhibiting ZEB2-related cellular pluripotency and invasiveness (Fig. 4K).

### Higher expression of ZEB2 was related to a poorer prognosis in HGSOC patients

**Fig. 5:**
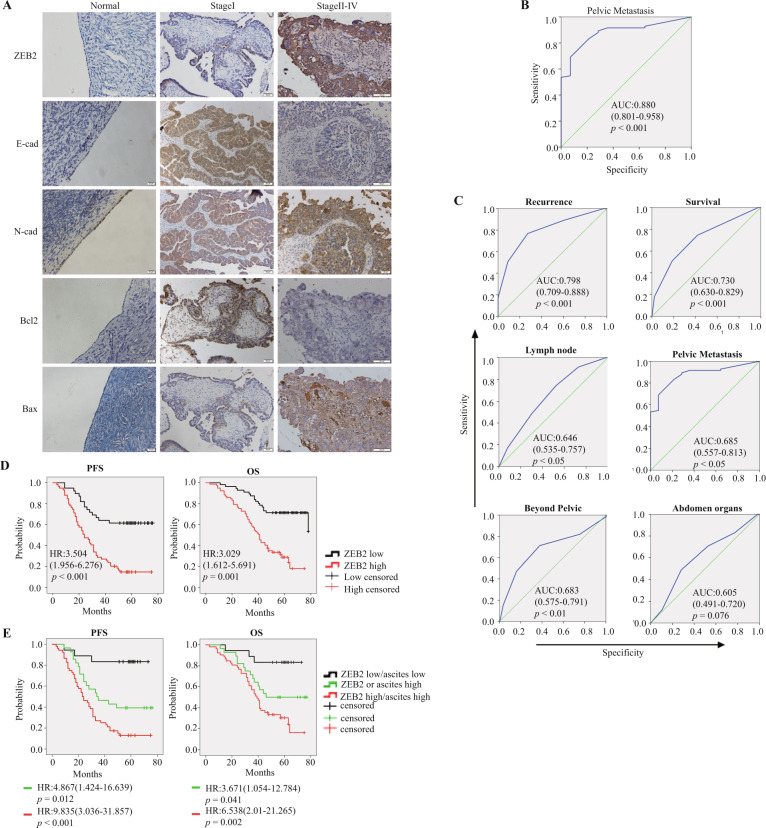
High ZEB2 expression in tumour tissue predicted peritoneal metastasis and a poorer outcome of HGSOC. **A** Representative IHC staining of ZEB2, E-cad, N-cad, Bcl2 and Bax in normal OSE samples and stage I and stages II–IV HGSOC tumour samples. Scale bar = 20 (normal ovaries), 50 or 100 μm (stage I and stages II–IV). **B** ROC curve of the ascites volume for pelvic metastasis in HGSOC patients. Cut-off value = 75 ml. **C** ROC curve of the IHC ZEB2 scores for metastasis in the pelvis, beyond the pelvis or in abdominal organs or lymph nodes; and the recurrence and survival status of HGSOC patients. **D** Kaplan–Meier analysis of the survival (PFS and OS) of HGSOC patients between the IHC ZEB2 high and low cohorts. **E** Kaplan–Meier analysis of the survival (PFS and OS) of HGSOC patients between the ZEB2^high^ascite^high^, ZEB2^low^ascite^low^ and ZEB2^high^ or ascite^high^ groups. *p < 0.05, **p < 0.01, ***p < 0.001.

To verify the prognostic potential of ZEB2 in HGSOC patients, the expression profiles of ZEB2, E-cad, N-cad, Bcl2 and Bax and the survival information of 98 HGSOC patients were analysed. Among them, 7 patients were in stage I/II, and 91 patients were in stage III/IV (Table S1). Normal ovary surface epithelium (OSE) obtained from the patients diagnosed with hysteromyoma was used as a control. Compared to those in OSE controls, ZEB2, N-cad and Bax were higher in HGSOC tumour tissues, while E-cad and Bcl2 were lower, and these changes were amplified with progressing stage (Fig. 5A, Tables S2 and S3).

Because intraperitoneal metastasis is a major unfavourable prognostic factor in HGSOC patients, we assessed the value of ZEB2 in predicting intraperitoneal metastasis using correlation analysis and receiver operating characteristic (ROC) curves. The expression of ZEB2 was consistent with the ascites volume at the cut-off of 75 ml (Fig. 5B). In addition, ZEB2 expression was significantly related to the extent of intraperitoneal metastasis (present beyond the pelvis vs absent), the infiltration of lymph nodes, and the CA125 index at the pre-treatment stage (Table S1), which were also statistically significant related to the status of recurrence and survival according to the area under the ROC curve (Fig. 5C).

We thus sought to investigate the prognostic value of ZEB2 by univariate Cox’s proportional hazard model. Higher expression of ZEB2 was evidently correlated with worse progression-free survival (PFS) (hazard ratio (HR) = 3.504, *p* < 0.001) and overall survival (OS) (HR = 3.029, *p* < 0.001) and with clinical variables associated with aggressive disease behaviour (Fig. 5D, Tables 1 and 2). The multivariate Cox proportional hazard model revealed that ZEB2 expression and beyond-the-pelvis metastasis were significantly associated with PFS and OS (Tables 1 and 2). In addition, compared to those in the ZEB2^high^, ascites^high^ (≥75 ml) or ZEB2^low^/ascites^low^ groups (<75 ml), the PFS and OS rates in the ZEB2^high^/ascites^high^ group were significantly worse, which was noteworthy and adequate to prove that integrating ZEB2 with ascites status improves the predictive ability of the markers (Fig. 5E, Tables 1 and 2).

**Table 1:**
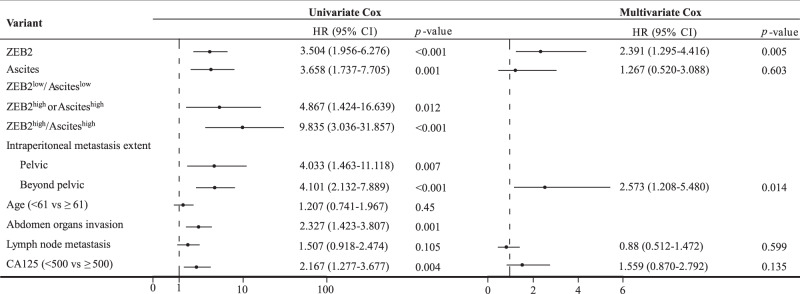
Univariate and multivariate Cox analysis of PFS in HGSOC patients.

**Table 2:**
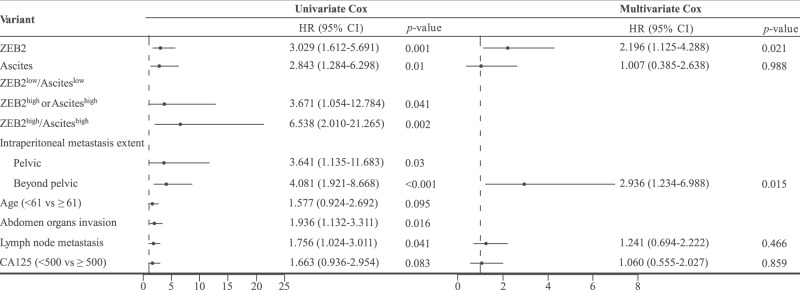
Univariate and multivariate Cox analysis of OS in HGSOC patients.

## Discussion

Malignant ascites is an important clinical outcome of HGSOC invasion [28–30] and promotes the dissemination of cancer cells from the primary tumour mass to the peritoneal cavity. However, intraperitoneal metastasis is not a simple tumour cell shedding and dissemination. Heterogeneity was observed intratumourally and between the primary tumour and ascitic spheroids, suggesting monophyletic spread in malignant ascites [31]. It has been indicated that CSLCs, with high differentiation and proliferation capacity [32], may play an important role in peritoneal metastasis. However, the underlying mechanism remains largely unknown.

Consistent with a previous study [33], we found that the percentage of ovarian CSLCs (not only SP cells but also CD133^+^ cells) was higher in ascitic fluid than in primary tumours. Since CSLCs are more able to resist anoikis after detaching from primary tumours [34], our results provide evidence of the contribution of CSLCs to the abdominal metastasis of HGSOC.

The process by which EOC-CSLCs detach from the primary tumour mass triggers dynamic EMT changes in cellular re-organisation from an epithelial-to-mesenchymal phenotype [17]. Malignant ascites-related pathogenesis is associated with upregulation of EMT signalling to promote tumour cell invasion in both EOCs and gastric cancers [35, 36]. Six steps constitute the metastasis cascade, namely, dissociation, migration, intravasation, extravasation, reestablishment and eventual formation of secondary tumours in distant organs, during which EMT and mesenchymal–epithelial transition occur [16]. In searching for the key transcript regulating EOC-CSLC-initiated peritoneal metastasis, we found that the difference in ZEB2 between ascites and primary tumours was more distinguishing than that of other transcripts. We additionally screened the expression of inhibitory miR-200 family members and discovered that miR-200a is the key interrupter that post-transcriptionally counters ZEB function. Thus, the role of the reciprocal miR-200a/ZEB2 loop in EMT-related tumorigenesis and EOC-CSLC-initiated metastasis was dissected in this study.

In addition to finding that the cells in ascites both harboured more CSLCs and expressed higher ZEB2, we found that ZEB2 was increased in CD133^+^ cells. Silencing ZEB2 not only reduced the CSLC percentages but also impacted the self-renewal and multipotency of EOC-CSLCs. The EMT-enhanced pathologic process of EOCs, which mediates invasive capacity, cellular apoptosis resistance and in vivo tumorigenicity, was impaired in ZEB2^-/-^ cells compared with wild-type ZEB2 cells. The introduction of miR-200a-mimic/inhibitor into malignant cells provided concordant information on the miR-200a/ZEB2 loop [37]. Our results further indicated that miR-200a/ZEB2 are more linked with CSLCs than with NCSLCs and play an important role in inducing the abdominal metastasis of EOCs.

From the perspective of translational application, ZEB2 has been reported as an adverse prognostic marker in some tumours, such as breast cancer [38], bladder carcinoma [39] and glioma [40]. We thus retrospectively analysed 98 HGSOC patients to clarify the relationship between ZEB expression and clinical outcome. We found that the expression of ZEB2 was associated with the OS of HGSOC patients, which is consistent with a previous study [41]. In addition, the integrity analysis of ZEB2 and ascites volume brought in apparent higher HRs for PFS and OS in the univariate Cox analysis. Higher ZEB2 expression or ZEB2^high^/ascites^high^ status was indicated as a risk indicator for recurrence according to their higher HRs for PFS.

Collectively, the relationship between ZEB2 expression and ovarian CSLC-initiated metastasis is shown in Fig. 4K. Depending on the activation of ZEB2 or the suppression of miR-200a, EMT occurs in the CSLC subpopulation and leads to the repression of epithelial characteristics and acquisition of mesenchymal features. EOC metastasis encompasses a series of pathologic process, including self-renewal, invasiveness, anti-anoikis and tumorigenesis, in which EOCs detach from the primary loci via the EMT programme and proliferate in distant organs to ultimately form intraperitoneal metastases. Our study improves the understanding of the regulatory mechanisms related to the miR-200a/ZEB2 loop in ovarian CSLC-initiated peritoneal metastasis, which may be a potential marker for prognostic evaluation and therapeutic intervention.

## Materials and methods

### Patient samples

Ascites and primary tumours were obtained from 23 HGSOC patients at the time of debulking surgery at Shanghai Cancer Center, Fudan University, from 2013 to 2014 (Table S4); the patients had a median age of 67 (54–78) years old. All patients were in stage III/IV. In addition, 98 HGSOC patients (7 patients in stage I/II; 91 patients in stage III/IV) who received surgery at Obstetrics & Gynecology Hospital, Fudan University, from 2009 to 2012 were included in our retrospect cohort with a median follow-up of 60 (26–77) months. The pathological characteristics of these patients are listed in Table S5. Control OSE tissues were obtained from 82 patients who were diagnosed with hysteromyoma undergoing post-unilateral uterine adnexectomy, who had a median age of 51 (45–55) years old.

All the patients’ diagnoses in this study were pathologically confirmed, and no patients received pre-operative chemotherapy or had a family history of breast or ovarian cancer. The proposal for this study was approved by the Institutional Review Board of Obstetrics & Gynecology Hospital, Fudan University, (2016-29), and informed consent was obtained from all subjects.

### Cell lines and cell cultivation

Human EOC cell line SKOV3 cells were obtained from American Type Culture Collection. SKOV3-IP and HEY cells were provided by Dr. Yinhua Yu, Obstetrics & Gynecology Hospital, Fudan University. HEY-A8 cells were provided by Professor Gong Yang, Shanghai Cancer Center, Fudan University. HSFs were purchased from Shanghai Fuheng Biological Technology Co. The lines HEY-A8 and SKOV3-IP were the highly metastatic daughter lines of HEY and SKOV3, respectively [42, 43]. HSF cells were maintained in Dulbecco’s modified Eagle’s medium (HyClone) with 10% FBS, while the remaining lines were cultivated in Roswell Park Memorial Institute 1640 (RPMI 1640, HyClone) containing 10% FBS. All the cell lines were tested free of mycoplasma contamination.

### Cell collection from ascites and primary tumours

The cells were isolated from ascitic fluid by 50% v/v Percoll density gradient centrifugation. The tumour specimens, especially the cauliflower-like cancer tissue, were washed with sterile PBS to remove blood clots, necrotic and fatty tissue and digested with 0.2 mg/ml collagenase type I (Sigma, USA) in MCDB/M199 at 37 °C for 4 h, and single cells were obtained by passing the samples through 70-μm and 40-μm cell strainers.

A time-dependent method was used to discard tumour stromal cells [44]. Cultivated tumour cells reaching 80–90% confluence were trypsinised. When the surrounding fibroblasts disaggregated entirely, the supernatant was discarded, and the remaining ascites- or primary tumour-derived colonies were continuously cultured. The tumour characteristics were assessed by staining with CA125 and HE4. All the cells applied in subsequent experiments were cultured for 2–3 weeks; the cells were purified each time when they were passaged during this time period.

### Flow cytometry analysis and cell sorting

For the analysis of cell surface markers, cells were trypsinised and incubated for 20 min with murine anti-human antibodies, the information of antibodies was listed in Table S5. The expression of intracellular markers (Bcl2 and active caspase-3) was detected by incubating the permeabilised cells with Alexa Fluor 647-conjugated murine anti-human Bcl2 or active caspase-3 (Table S5).

For apoptosis analysis, an annexin V-APC/7-AAD apoptosis detection kit (BD Pharmingen, USA) was used following the manufacturer’s instructions. We took the total proportion of annexin V-positive cells (quadrants II and III) as the apoptotic rate, regardless of the 7-AAD status. FACS analysis was performed on a FACS Calibur (BD Biosciences, USA), and data analysis was performed using FlowJo Software (TreeStar, USA).

CD133^+^ and SP cells were sorted for the analysis of CSLC. The staining procedure of SP cells was described previously [26]. Hoechst 33342-effluxing cells were analysed and sorted by ultraviolet laser cytometry (FACS MoFlo, Beckman Coulter, USA), while CD133^+^ cells were sorted on a FACS Aria III (BD Biosciences, USA). Data analysis was performed using Summit Software (Beckman Coulter, USA).

### Lentiviral transduction

The oligo sequences of the ZEB2-specific shRNA (shZEB2) duplex and NC duplex were 5′-ACCAUGAAUAGUAAUUUAATT-3′ and 5′-GGCTACGTCCAGGAGCGCA-3′, respectively. Lentiviral vectors encoding short hairpin RNAs were generated by inserting shZEB2 or NC into the U6-MCS-Ubi-EGFP-vector and hU6-MCS-Ubiquitin-Luc-firefly IRES-puromycin-vector (both from GeneChem lnc, Shanghai, China), respectively. The transfected GFP^+^ cells were selected by fluorescence-activated cell sorting or purified by the addition of 2 μg/ml puromycin to the culture media.

### Transfection of miR mimics and inhibitors

Cells (3 × 10^5^ per well in a six-well plate) were cultured to reach a confluency of 60–80%. A mixture of miR-200a-mimic (10 nM, Qiagen, Germany, 5′-UAACACUGUCUGGUAACGAUGU-3′) or inhibitor (50 nM, Qiagen, Germany, 5′-UAACACUGUCUGGUAACGAUGU-3′) with HiPerFect Transfection Reagent (Qiagen, Germany) was applied to transfect primary tumour cells and freshly sorted CSLCs and non-CSCLs according to the manufacturer’s instructions. After culturing for 24 or 48 h, the transfected tumour cells were harvested for the collection of RNAs or proteins. Serial transfection (every 3 days) was performed for the analysis of cell morphology and colony forming ability.

### RT-PCR analysis

Total RNA was extracted using TRIzol reagent (Invitrogen, USA) and reverse transcribed into cDNA using the PrimeScript reverse transcription kit (Takara, Japan). RT-PCR was subsequently performed in triplicate using the Premix SYBR green PCR system (Takara, Japan) on an ABI Step One Plus RT-PCR machine (Applied Biosystems, USA). The oligonucleotide primers are listed in Table S6. The expression of miR-200s (miR-200a, miR-200b, miR-200c, miR-141 and miR-429) relative to that of small nucleolar RNA U6 was determined by using the miScript PCR System (Qiagen, Germany) according to the manufacturer’s instructions.

### Western blotting analysis

The protein was resolved by 10% SDS-PAGE and then transferred onto PVDF membranes, which were treated sequentially in blocking solution and then incubated with primary antibodies overnight at 4 °C and secondary antibodies for 1 h at room temperature. The information of antibodies was listed in Table S5. After incubation with enhanced chemiluminescence reagents, the membranes were visualised under an Image Quant LAS 4000 (GE Healthcare, USA).

### Colony formation assay

Two hundred cells/well were seeded in a six-well plate at 37 °C for 12 days, and the medium was replaced with fresh complete medium every 4 days. The attached cells were fixed and subjected to 1% crystal violet staining to detect colonies consisting of at least 50 cells using ImageJ Software (Rawak Software, Germany).

### Transwell invasion assay

Sixty microlitres of cold-melted Matrigel (BD, USA) at a 1:5 dilution was coated and solidified on a Transwell membrane with 8-µm pores (Corning Costar, USA) at 37 °C for 5 h. The cells were resuspended in serum-free RPMI 1640 at a concentration of 1–6 × 106 cells/ml. One hundred microlitres of the cell suspension was plated on the Matrigel-covered upper chamber, while 600 μl RPMI 1640 medium containing 30% FBS was added to the lower chamber. After culturing at 37 °C for 24–36 h, the membranes were stained with 0.1% crystal violet and visualised under a Nikon upright microscope.

### In vivo xenograft transplantation

Female NOD/SCID mice (5 weeks old) were purchased from Shanghai SLAC Laboratory Animal Co. Ltd, and were randomised into four groups. Each group included six mice without blinding controls. Luciferase-expressing CD133^+^ or CD133^−^ cells derived from the shZEB2- or NC-transfected HEY-A8 cells (5 × 10^5^) were injected into the mice intraperitoneally. Luciferin (Promega, USA) was used to ensure the detection of the emitted light in an In-Vivo Xtreme II Imaging System (Bruker, Germany). In addition, the metastatic tumours in the abdominal cavity were surgically removed and formalin-fixed for immunohistochemistry (IHC) staining. The animal experiment procedure was reviewed and approved by the Ethical Committee of Animal Experiments of the School of Pharmacy, Fudan University (2015-05-FCKYY-JH-01).

### In situ immunofluorescence and IHC

Cells cultured on chamber slides were fixed and permeabilised in PBS containing 0.2% Triton X-100 for 10 min. Murine anti-human antibodies, including Alexa Fluor 488-conjugated anti-E-cad (1:40, #560061, BD Pharmingen, USA) and Alexa Fluor 647-conjugated anti-VIM (1:100, #MA5-11883-A647, Thermo Fisher Scientific, USA), were added to the slides and incubated overnight at 4 °C, while a goat anti-mouse IgG1 conjugated with Alexa Fluor 488 (1:200, # A-21121, Invitrogen, USA) was used as the secondary antibody. The fluorescence images were investigated under a Leica TCS SP5 confocal microscope (Leica Microsystems, Germany) with DAPI co-staining.

Paraffin-embedded tissues were obtained from the primary tumours or normal ovaries at initial surgery. Ten millimolar preheated citrate buffer (pH 6) and 3% H_2_O_2_ were used to retrieve antigens and block non-specific peroxidase activity. The slides were incubated with antibodies at 4 °C overnight. The information of antibodies was listed in Table S5. Secondary antibody was subsequently incubated for 30 min, and 3,3′-diaminobenzidine was used for detection using the GTVision^TM^ II Detection System (Gene Tech, USA). All the 98 HGSOC patients were detected ZEB2 expression in tumour tissues by IHC, and expression of N-cad, E-cad, Bax and Bcl2 was determined in 64 cases from 2010 to 2012.

Protein expression was scored by two independent observers according to previously published methodology [45]. Briefly, protein expression was quantified using a scoring system defined as signalling intensity (A) × the percentage of positive cells (B), in which 0, 1, 2, 3 in (A) represent no staining, weak staining and strong staining, and 0–3 in (B) represent 0%, 1–25%, 25–50% and >50% positively stained cells, respectively. Final scores of 1–3 indicated proteins with low (negative) expression, and final scores of 4–9 indicated proteins with high (positive) expression.

### Statistical analysis

Statistical analysis was performed with SPSS 22.0 (IBM, USA) and GraphPad Prism 4.0 (GraphPad Software, USA). For the cellular data, the significance of the difference in the mean value was determined using a two-tailed Student’s *t* test. Chi-square and Spearman rank correlation tests were conducted to assess the association between ZEB2 and pathological parameters. ROC curves were applied to evaluate the predictive value of ZEB2 in terms of clinical features. Survival curves were drawn using the Kaplan–Meier method. OS and PFS were determined from the date of diagnosis to the date of progression/death or date last seen. Univariate and multivariate Cox proportional hazards regression analyses were employed to investigate the prognostic value and HRs of ZEB2 and a series of variables. The results are presented as the mean ± SD of at least three independent experiments. Differences with a *p* value of < 0.05 were considered significant.

## Supplementary information

Supplementary material

## References

[1] Siegel RL, Miller KD, Jemal A (2018). Cancer statistics, 2018. CA Cancer J Clin.

[2] Hooda J, Novak M, Salomon MP, Matsuba C, Ramos RI, MacDuffie E (2019). Early loss of histone H2B monoubiquitylation alters chromatin accessibility and activates key immune pathways that facilitate progression of ovarian cancer. Cancer Res.

[3] Zheng T, Qiu J, Li C, Lin X, Tang X, Hua K (2019). Long noncoding RNA LINC00673 promotes the proliferation and metastasis of epithelial ovarian cancer by associating with opioid growth factor receptor. Onco Targets Ther.

[4] Torre LA, Trabert B, DeSantis CE, Miller KD, Samimi G, Runowicz CD (2018). Ovarian cancer statistics, 2018. CA Cancer J Clin.

[5] Saini U, Naidu S, Elnaggar AC, Bid HK, Wallbillich JJ, Bixel K (2017). Elevated STAT3 expression in ovarian cancer ascites promotes invasion and metastasis a potential therapeutic target. Oncogene..

[6] Kim S, Kim B, Song YS (2016). Ascites modulates cancer cell behavior, contributing to tumor heterogeneity in ovarian cancer. Cancer Sci.

[7] Webb TJ, Li X, Giuntoli RL, Lopez PH, Heuser C, Schnaar RL (2012). Molecular identification of GD3 as a suppressor of the innate immune response in ovarian cancer. Cancer Res.

[8] Matte I, Lane D, Laplante C, Rancourt C, Piché A. Profiling of cytokines in human epithelial ovarian cancer ascites. Am J Cancer Res. 2012;2:566–80. http://www.pubmedcentral.nih.gov/articlerender.fcgi?artid=PMC3433103. PMC3433103 22957308

[9] Shi J, Huo R, Li N, Li H, Zhai T, Li H (2019). CYR61, a potential biomarker of tumor inflammatory response in epithelial ovarian cancer microenvironment of tumor progress. BMC Cancer.

[10] Ignacio RMC, Lee E-S, Wilson AJ, Beeghly-Fadiel A, Whalen MM, Son D-S (2018). Chemokine network and overall survival in TP53 wild-type and mutant ovarian cancer. Immune Netw.

[11] Lourenço AR, Roukens MG, Seinstra D, Frederiks CL, Pals CE, Vervoort SJ (2020). C/EBPa is crucial determinant of epithelial maintenance by preventing epithelial-to-mesenchymal transition. Nat Commun.

[12] Wu Q, Li G, Wen C, Zeng T, Fan Y, Liu C (2020). Monoubiquitination of p120-catenin is essential for TGFbeta-induced epithelial-mesenchymal transition and tumor metastasis. Sci Adv.

[13] Jolly MK, Somarelli JA, Sheth M, Biddle A, Tripathi SC, Armstrong AJ (2019). Hybrid epithelial/mesenchymal phenotypes promote metastasis and therapy resistance across carcinomas. Pharm Ther.

[14] Yeung KT, Yang J (2017). Epithelial-mesenchymal transition in tumor metastasis. Mol Oncol.

[15] Tripathi S, Levine H, Jolly MK. The physics of cellular decision making during epithelial-mesenchymal transition. Annu Rev Biophys. 2020. 10.1146/annurev-biophys-121219-081557. 10.1146/annurev-biophys-121219-081557 31913665

[16] Yang J, Antin P, Berx G, Blanpain C, Brabletz T, Bronner M (2020). Guidelines and definitions for research on epithelial–mesenchymal transition. Nat Rev Mol Cell Biol.

[17] Loret N, Denys H, Tummers P, Berx G. The role of epithelial-to-mesenchymal plasticity in ovarian cancer progression and therapy resistance. Cancers. 2019;11. 10.3390/cancers11060838. 10.3390/cancers11060838 PMC6628067 31213009

[18] Scott CL, T'jonck W, Martens L, Todorov H, Sichien D, Soen B (2018). The transcription factor ZEB2 is required to maintain the tissue-specific identities of macrophages. Immunity.

[19] Liu C, Yang F (2015). Akt2/ZEB2 may be a biomarker for exfoliant cells in ascitic fluid in advanced grades of serous ovarian carcinoma. Tumour Biol.

[20] Song N, Liu H, Ma X, Zhang S (2016). Placental growth factor promotes ovarian cancer cell invasion via ZEB2. Cell Physiol Biochem.

[21] Choi P-W, Ng S-W (2017). The functions of MicroRNA-200 family in ovarian cancer: beyond epithelial-mesenchymal transition. Int J Mol Sci.

[22] Shepherd TG, Thériault BL, Campbell EJ, Nachtigal MW (2006). Primary culture of ovarian surface epithelial cells and ascites-derived ovarian cancer cells from patients. Nat Protoc.

[23] Den Ouden JE, Zaman GJR, Dylus J, Van Doornmalen AM, Mulder WR, Grobben Y (2020). Chemotherapy sensitivity testing on ovarian cancer cells isolated from malignant ascites. Oncotarget.

[24] Yamanoi K, Baba T, Abiko K, Hamanishi J, Yamaguchi K, Murakami R (2019). Acquisition of a side population fraction augments malignant phenotype in ovarian cancer. Sci Rep.

[25] Baba T, Convery PA, Matsumura N, Whitaker RS, Kondoh E, Perry T (2009). Epigenetic regulation of CD133 and tumorigenicity of CD133+ ovarian cancer cells. Oncogene.

[26] Jiangiang H, Lin X, Liu Y, Gong W, Ma X, Yu Y (2012). Transformation of epithelial ovarian cancer stemlike cells into mesenchymal lineage via EMT results in cellular heterogeneity and supports tumor engraftment. Mol Med.

[27] Liu C, Hu W, Li L-L, Wang YX, Zhou Q, Zhang F (2018). Roles of miR-200 family members in lung cancer: more than tumor suppressors. Future Oncol.

[28] Kotrbová A, Ovesná P, Gybel' T, Radaszkiewicz T, Bednaříková M, Hausnerová J (2020). WNT signaling inducing activity in ascites predicts poor outcome in ovarian cancer. Theranostics.

[29] Han Q, Huang B, Huang Z, Cai J, Gong L, Zhang Y (2019). Tumor cellfibroblast heterotypic aggregates in malignant ascites of patients with ovarian cancer. Int J Mol Med.

[30] Alberto-Aguilar DR, Hernandez-Ramirez VI, Osorio-Trujillo JC, Gallardo-Rincon D, Toledo-Leyva A, Talamas-Rohana P (2019). Ascites from ovarian cancer induces novel fucosylated proteins. Cancer Microenviron.

[31] Kim S, Kim S, Kim J, Kim B, Kim SI, Kim MA (2018). Evaluating tumor evolution via genomic profiling of individual tumor spheroids in a malignant ascites. Sci Rep.

[32] Lytle NK, Barber AG, Reya T (2018). Stem cell fate in cancer growth, progression and therapy resistance. Nat Rev Cancer.

[33] Mo L, Bachelder RE, Kennedy M, Chen PH, Chi JT, Berchuck A (2015). Syngeneic murine ovarian cancer model reveals that ascites enriches for ovarian cancer stem-like cells expressing membrane GRP78. Mol Cancer Ther.

[34] Aslan B, Monroig P, Hsu M-C, Pena GA, Rodriguez-Aguayo C, Gonzalez-Villasana V (2015). The ZNF304-integrin axis protects against anoikis in cancer. Nat Commun.

[35] Hu Y, Qi C, Liu X, Zhang C, Gao J, Wu Y (2019). Malignant ascites-derived exosomes promote peritoneal tumor cell dissemination and reveal a distinct miRNA signature in advanced gastric cancer. Cancer Lett.

[36] Pakułaakuła M, Mikuła-Pietrasik J, Witucka A, Kostka-Jeziorny K, Uruski P, Moszyński R (2019). The epithelial-mesenchymal transition initiated by malignant ascites underlies the transmesothelial invasion of ovarian cancer cells. Int J Mol Sci.

[37] Zhao Y-X, Sun Y-Y, Huang A-L, Li XF, Huang C, Ma TT (2018). MicroRNA-200a induces apoptosis by targeting ZEB2 in alcoholic liver disease. Cell Cycle.

[38] Gu S, Chu C, Chen W, Ren H, Cao Y, Li X (2019). Prognostic value of epithelial-mesenchymal transition related genes: SLUG and QKI in breast cancer patients. Int J Clin Exp Pathol.

[39] Moussa RA, Khalil EZI, Ali AI (2019). Prognostic role of epithelial-mesenchymal transition markers “E-cadherin, beta-catenin, ZEB1, ZEB2 and p63” in bladder carcinoma. World J Oncol.

[40] Song Q, Pang H, Qi L, Liang C, Wang T, Wang W (2019). Low microRNA-622 expression predicts poor prognosis and is associated with ZEB2 in glioma. Onco Targets Ther.

[41] Yan Z, Tian X, Wang R, Cheng X, Mi J, Xiong L (2017). Title prognosis significance of ZEB2 and TGF-beta1 as well as other clinical characteristics in epithelial ovarian cancer. Int J Gynecol Cancer.

[42] Ahluwalia A, Hurteau JA, Bigsby RM, Nephew KP (2001). DNA methylation in ovarian cancer. II. Expression of DNA methyltransferases in ovarian cancer cell lines and normal ovarian epithelial cells. Gynecol Oncol.

[43] Yu D, Wolf JK, Scanlon M, Price JE, Hung MC (1993). Enhanced c-erbB-2/neu expression in human ovarian cancer cells correlates with more severe malignancy that can be suppressed by E1A. Cancer Res.

[44] Zhang Y, An J, Liu M, Li N, Wang W, Yao H (2020). Efficient isolation, culture, purification, and stem cell expression profiles of primary tumor cells derived from uterine cervical squamous cell carcinoma. Am J Reprod Immunol.

[45] Yang D, Sun Y, Hu L, Zheng H, Ji P, Pecot CV (2013). Integrated analyses identify a master microRNA regulatory network for the mesenchymal subtype in serous ovarian cancer. Cancer Cell.

