# Supplementary material for: ZEB2 facilitates peritoneal metastasis by regulating the invasiveness and tumorigenesis of cancer stem-like cells in high-grade serous ovarian cancers
Source: Oncogene. 2021 Jul 1;40(32):5131–41. doi: 10.1038/s41388-021-01913-3 (PMC8363099; doi:10.1038/s41388-021-01913-3)
Supplement: Supplementary file 1 — Supplementary material [file 41388_2021_1913_MOESM1_ESM.pdf]

Supplementary Information for:

ZEB2 Facilitates Peritoneal Metastasis by Regulating the Invasiveness and Tumorigenesis of Cancer Stem-Like Cells in High-Grade Serous Ovarian Cancers

Running title: **ZEB2 regulates CSLCs to facilitate the metastasis of HGSOC**

Yiying Li, He Fei, Qiwang Lin, Fan Liang, Yanan You, Ming Li, Mengyao Wu, Ying Qu, Pengfei Li, Yan Yuan, Tong Chen, Hua Jiang

Table S1 to S6

Figures S1 to S4

Table S1. Relationship between ZEB2 expression and tumor characteristics

Variant	No. (%)	ZEB2				
		-	+	*p*-value	Odds Ratio*	95% CI*
**Age**						
< median = 61 y	53 (54.1)	21(39.6)	32 (60.4)	0.970	0.984	0.437-2.216
≥ median = 61 y	45 (45.9)	18 (40.0)	27 (60.0)			
**Ascites**						
<75ml	25 (25.5)	18 (72.0)	7 (28.0)	<0.001	6.367	2.320-17.473
≥75ml	73 (74.5)	21 (28.8)	52 (71.2)			
**Large tumor size**						
<5cm	30 (30.6)	10 (33.3)	20 (66.7)	0.385	0.672	0.274-1.651
≥5cm	68 (69.4)	29 (42.6)	39 (57.5)			
**Pelvic metastasis**						
Absent	14 (14.3)	8 (57.2)	6 (36.9)	0.152	2.280	0.724-7.182
Pelvic	84 (85.7)	31 (36.9)	53 (63.1)			
**Beyond pelvic metastasis**						
Absent	32 (32.7)	20 (62.5)	12 (37.5)	0.001	4.123	1.689-10.062
Beyond pelvic	66 (67.3)	19 (28.8)	47 (71.2)			
**Abdomen organs invasion**						
Absent	57 (58.2)	27 (47.4)	30 (52.6)	0.071	2.175	0.929-5.090
Present	41 (41.8)	12 (29.3)	29 (70.7)			
**Lymph node involvement**						
PN0	63 (64.3)	30 (47.6)	33 (52.4)	0.034	2.626	1.062-6.492
PN+	35 (35.7)	9 (25.7)	26 (74.3)			
**CA125 index***						
<35	1 (1.0)	1 (100)	0 (0)	0.025		
≥35, <500	39 (39.8)	21 (53.8)	18 (46.2)			
≥500	58 (59.2)	17 (29.3)	41 (70.7)			

* The parameters of odds ratio and 95% CI were calculated for the two-category data in chi-square test, which were not provided in the variables of CA125 index (three-category) here.

Table S2. Expression of ZEB2 in OSE and HGSOC tumors

Variant	Total	ZEB2		
		-	+	*p*-value
**OSE**	82	77 (93.9%)	5 (6.1%)	<0.001
**HGSOC**	98	39(39.8%)	59(60.2%)	

Table S3. Expression of E-cad, N-cad, Bcl2 and Bax in OSE and HGSOC tumors

Variant	Total	E-cad			N-cad			Bcl2			Bax		
		-	+	*p*-value	-	+	*p*-value	-	+	*p*-value	-	+	*p*-value
**OSE**	82	74 (90.2%)	8 (9.8%)	0.005	66 (80.5%)	16 (19.5%)	<0.001	78 (95.1%)	4 (4.9%)	<0.001	79 (96.3%)	3 (3.7%)	<0.001
**HGSOC**	64	46 (71.9%)	18 (28.1%)		24 (37.5%)	40 (62.5%)		37 (57.8%)	27 (42.2%)		19 (29.7%)	45 (70.3%)	

Table S4. Clinical characteristics of 23 cases of HGSOC for tumor cell culture

Variant	No. (%)
**All cases**	23
**Median Age (years, range)**	67 (54-78)
**Ascites**	
Yes	23 (100)
No	0 (0)
**FIGO Stage**	
I-II	0 (0)
III-IV	23 (100)
**Peritoneal carcinomatosis**	
Yes	23 (100)
No	0 (0)

Table S5. Primary antibody information for Western blotting, IHC and Flow Cytometry

Antibody	Concentration			Catalog number and Manufacturer
	Flow Cytometry	Western blotting	IHC	
PE/APC-conjugated anti-human CD133	1:20			# 130-118-061, Miltenyi Biotec, USA
Alexa Fluor 647 anti-human E-cad	1:40			# 560062, BD Pharmingen, USA
Alexa Fluor 647 anti-human N-cad	1:40			# 563434, BD Pharmingen, USA
Alexa Fluor 647 anti-human VIM	1:100			#MA5-11883-A647, Thermo Fisher Scientific, USA
APC-conjugated anti-human FN	1:100			# 563098, BD Pharmingen, USA
Alexa Fluor 647 anti-human Bcl2	1:50			# 563600, BD Pharmingen, USA
Alexa Fluor 647 anti-human active caspase-3	1:50			# 560626, BD Pharmingen, USA
Rabbit anti-human ZEB2 antibody		1:800	1:150	# ab138222, Abcam, USA
Mouse anti-human E-cad antibody		1:800	1:100	# MAB1838-SP, R&D Systems, USA
Mouse anti-human N-cad antibody		1:1000	1:100	# MAB13881-SP, R&D Systems, USA
Rabbit anti-human Bax antibody		1:1000	1:150	# ab32503, Abcam, USA
Rabbit anti-human Bcl2 antibody		1:1000	1:100	# ab32124, Abcam, USA
Rabbit anti-human HE4 antibody		1:1000		# ab200828, Abcam, USA
Rabbit anti-human VIM antibody		1:1000		# ab92547, Abcam, USA
Rabbit anti-human MMP9 antibody		1:800		# ab76003, Abcam, USA
Rabbit anti-human MMP2 antibody		1:800		# ab92536, Abcam, USA
Rabbit anti-human Snai1 antibody		1:1000		#3879T, Cell Signalling Technology, USA
Rabbit anti-human CA125 antibody		1:1000		# AF2386, Beyotime, China
Rabbit anti-GAPDH antibody		1:5000		# ab9485, Abcam, USA
Goat Anti-Rabbit IgG H&L (HRP)		1:5000	1:2000	# ab6721, Abcam, USA
Goat Anti-Mouse IgG H&L (HRP)		1:5000	1:2000	# ab6789, Abcam, USA

Table S6. Oligonucleotide sequences used for Realtime-PCR analysis

Genes names	Sense primers (5' to 3')	Genes names	Sense primers (5' to 3')
hZEB2-F	AAAACCTCGCCAAGAGTGTC	hMMP2-F	TGATCTTGACCAGAATACCATCGA
hZEB2-R	GAGGCGTAACACGTCAGTCC	hMMP2-R	TGATCTTGACCAGAATACCATCGA
hZEB1-F	AAGAATTCACAGTGGAGAGAAGCCA	hBax-F	CGACTGATGTCCCTGTCTCC
hZEB1-R	CGTTTCTTGC AGTTTGGGCATT	hBax-R	CACTCCCGCCACAAAGATG
hSnail-F	GGTTCTTCTGCGTACTGCT	hBcl2-F	AACATCGCCCTGTGGATGAC
hSnail-R	AGGGCTGCTGGAAGGTAAACTCTGG	hBcl2-R	AGAGTCTTCAGAGACAGCCAGGAG
hTwist1-F	CACGAGCGGCTCAGCTACGC	hNanog-F	CAAAGGCAAACAACCCACTT
hTwist1-R	ACAATGACATCTAGGTCTCCGGCCC	hNanog-R	TCTGCTGGAGGCTGAGGTAT
hN-cadherin-F	AGCACAGTGGCCACCTACAAAG	hOct4-F	GACAACAATGAAAATCTTCAGGAGA
hN-cadherin-R	CAGCTCCTGGCCCAGTTACA	hOct4-R	TTCTGGCGCCGGTTACAGAACCA
hE-cadherin-F	TTCCCTCGACACCCGATTC	hβ-actin-F	GCCCTGAGGCACTCTTCCA
hE-cadherin-R	TAGGTGGAGTCCCAGGCGTA	hβ-actin-R	TTGCGGATGTCCACGTCA
hVimentin-F	TGACATTGAGATTGCCACCTACAG	hMMP9-F	TGGGCTACGTGACCTATGA
hVimentin-R	TCAACCGTCTTAATCAGAAGTGTCC	hMMP9-R	TCCACTCCTCCCTTTCCT
hMMP3-F	TTTCCAGGGATTGACTCAAA	hCA125-F	CCAGTCCTACATCTTCGGTTGT
hMMP3-R	AAGTGCCCATATTGTGCCTT	hCA125-R	AGGGTAGTTCCTAGAGGGAGTT
hMMP7-F	CCAAATCAACCATAGGTCCA	hHE4-F	CAAGAGTGCGTCTCGGACAG
hMMP7-R	AGCAGGCACACAGCACACA	hHE4-R	TTAATGTTCACCTGGGGGCA

Supplementary Figure legends

Supplementary Figure S1. Ascites Cells Derived from HGSOC Patients Expressed Higher Level of ZEB2 and Exhibited More Invasive Potential.

A. The relative mRNA levels of HE4 and CA125 in the cells derived from the ascites of 3 HGSOC patients and human skin fibroblasts.

B. The expression of HE4 and CA125 in the cells derived from the ascites of 3 HGSOC patients and human skin fibroblasts were analyzed by western blotting.

C. The relative mRNA levels of E-cad, N-cad, VIM and FN in ascites cells derived from HGSOC patients compared to paired primary tumor cells.

D. The relative mRNA levels of ZEB2, ZEB1, Snail and Twist1 in ascites cells derived from HGSOC patients compared to paired primary tumor cells.

E. The relative mRNA levels of MMP9, MMP2, MMP3 and MMP7 in ascites cells derived from HGSOC patients compared to paired primary tumor cells.

F. The expression of MMP9 and MMP2 in paired ascites cells and primary tumor cells derived from 3 HGSOC patients was analyzed by western blotting. The western blotting bands were quantified and normalized by GAPDH to indicate relative levels of MMP9 and MMP2.

The data represented the mean ± SD. Each experiment was repeated independently at least three times. * $p < 0.05$, ** $p < 0.01$, *** $p < 0.001$.

Supplementary Figure S2. ZEB2 was Upregulated in EOC-CSLCs and Correlated with their Mesenchymal Feature and Invasive Potential

A. The expression of MMP2 and MMP9 in SKOV3, HEY, and their daughter lines SKOV3-IP, HEY-A8 were analyzed by western blotting.

B. Expression of MMP9 and MMP2 in SKOV3, HEY, and their daughter lines SKOV3-IP, HEY-A8 were analyzed by RT-PCR.

C. The expression of N-cad, VIM and E-cad in SKOV3, HEY, and their daughter lines SKOV3-IP, HEY-A8 was analyzed by western blotting.

D. Dot plot (left panel) and grouped percentages (right panel) of VIM expression in SKOV3, SKOV3-IP, HEY and HEY-A8 cells were analyzed by flow cytometry analysis.

E. Dot plot (left panel) and grouped percentages (right panel) of N-cad expression in SKOV3, SKOV3-IP, HEY and HEY-A8 cells were analyzed by flow cytometry analysis.

The data represented the mean ± SD. Each experiment was repeated independently at least three times. * $p < 0.05$, ** $p < 0.01$, *** $p < 0.001$.

Supplementary Figure S3. ZEB2 Silencing Repressed the Pluripotency, Invasiveness and Anti-apoptosis of EOC-CSLCs.

A. Expression of ZEB2 in shZEB2 or NC transfected SKOV3-IP and HEY-A8 cell lines were analyzed by RT-PCR and normalized to the NC-transfected cells (=1).

B. The expression of ZEB2 in shZEB2 or NC transfected SKOV3-IP and HEY-A8 cell lines were analyzed by western blotting.

C. Expression of Nanog and Oct4 in shZEB2 or NC transfected SKOV3-IP and HEY-A8 cell lines were analyzed by RT-PCR and normalized to the NC-transfected cells (=1).

D. Expression of N-cad and VIM in shZEB2- or NC-transfected SKOV3-IP and HEY-A8 cells was analyzed by western blotting.

E. FACS analysis of Annexin V in $CD133^{+}/CD133^{-}$ cells from shZEB2/NC transfected HEY-A8 cells. Data were showed as dot plot (left panel) and grouped percentage of apoptotic cells (right panel).

The data represented the mean ± SD. Each experiment was repeated independently at least three times. * $p < 0.05$, ** $p < 0.01$, *** $p < 0.001$.

Supplementary Figure S4. MiR-200a Impacted on the ZEB2 expression and anti-apoptosis of EOC-CSLCs

A. The relative mRNA of miR-200a in miR200a-mimic/inhibitor transfected $CD133^{+}/CD133^{-}$ cells from HEY-A8 was compared to their NC counterparts.

B. The relative mRNA of ZEB2 in miR200a-mimic/inhibitor transfected $CD133^{+}/CD133^{-}$ cells from HEY-A8 was compared to their NC counterparts.

C. Western blotting analysis of Bcl2 in mimic/inhibitor transfected SKOV3-IP and HEY-A8 cells.

D. The relative mRNA of Bcl2 and Bax in mimic/inhibitor transfected $CD133^+$/$CD133^-$ cells from HEY-A8 was compared to their NC counterparts. The data represented the mean ± SD. Each experiment was repeated independently at least three times. * $p < 0.05$, ** $p < 0.01$, *** $p < 0.001$.

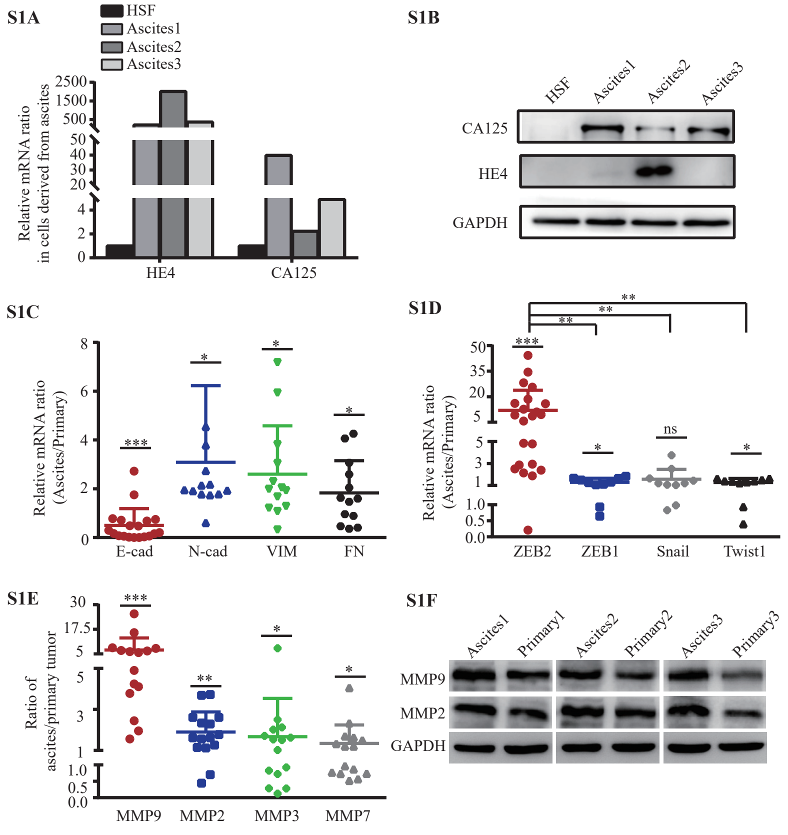

S1A
HSF
Ascites1
Ascites2
Ascites3
Relative mRNA ratio in cells derived from ascites
2500
1500
500
50
40
30
20
4
2
0
HE4
CA125
S1B
HSF
Ascites1
Ascites2
Ascites3
CA125
HE4
GAPDH
S1C
Relative mRNA ratio (Ascites/Primary)
8
6
4
2
0
***
*
*
*
E-cad
N-cad
VIM
FN
S1D
Relative mRNA ratio (Ascites/Primary)
**
**
**
***
*
ns
*
50
35
20
5
5
3
1
1.0
0.5
0.0
ZEB2
ZEB1
Snail
Twist1
S1E
Ratio of ascites/primary tumor
30
17.5
5
5
3
1
1.0
0.5
0.0
***
**
*
*
MMP9
MMP2
MMP3
MMP7
S1F
Ascites1
Primary1
Ascites2
Primary2
Ascites3
Primary3
MMP9
MMP2
GAPDH

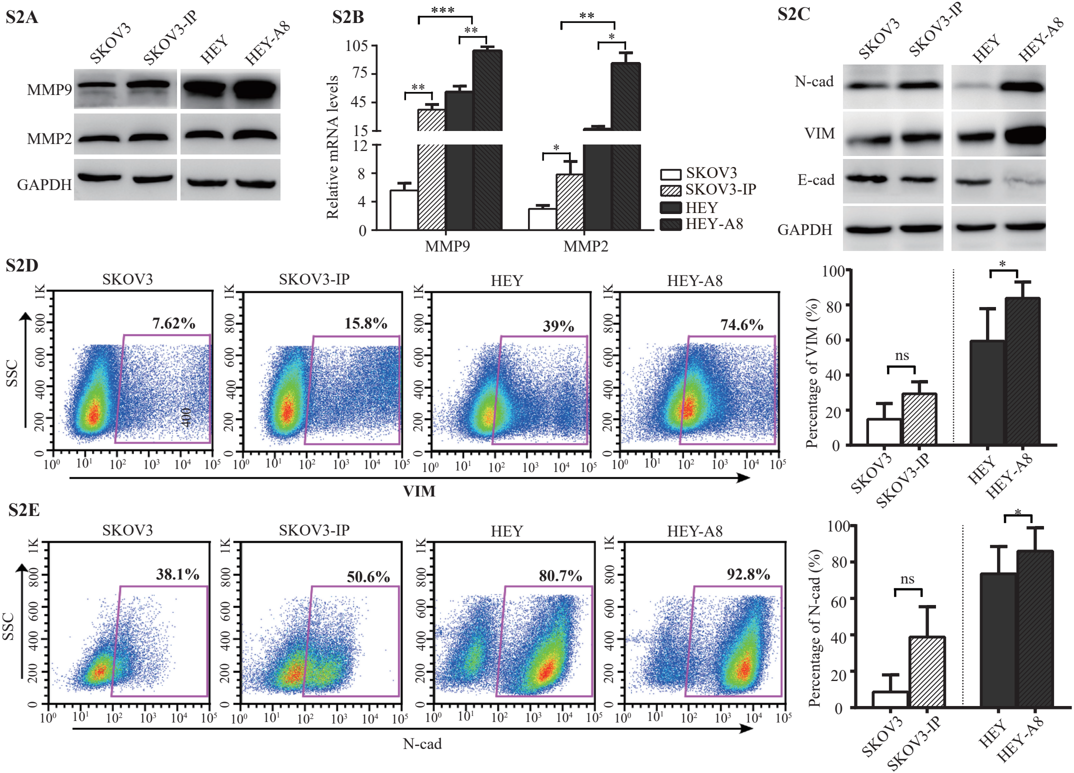

S2A
SKOV3
SKOV3-IP
HEY
HEY-A8
MMP9
MMP2
GAPDH
S2B
Relative mRNA levels
MMP9
MMP2
SKOV3
SKOV3-IP
HEY
HEY-A8
S2C
N-cad
VIM
E-cad
GAPDH
S2D
7.62%
15.8%
39%
74.6%
SSC
VIM
Percentage of VIM (%)
ns
S2E
38.1%
50.6%
80.7%
92.8%
N-cad
Percentage of N-cad (%)

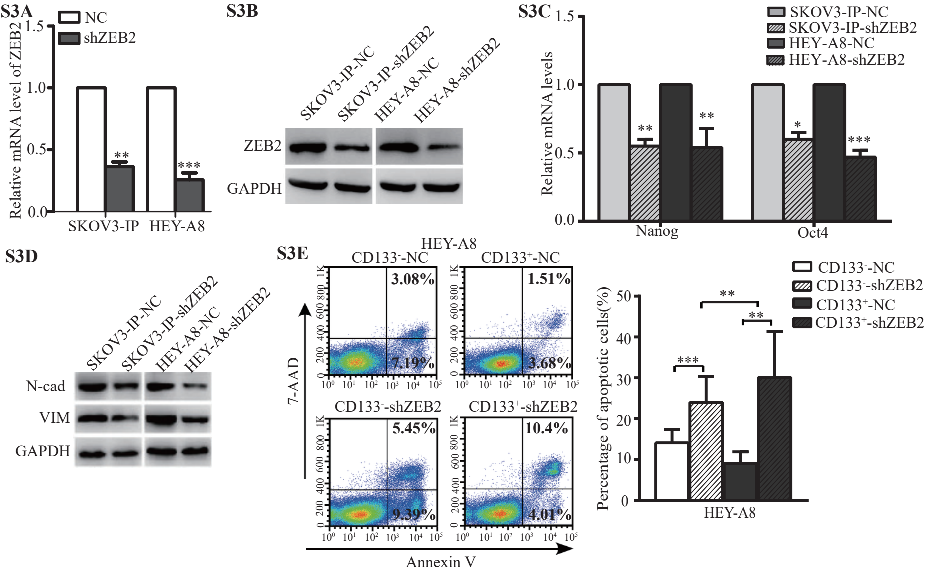
S3A
NC
shZEB2
Relative mRNA level of ZEB2
1.5
1.0
0.5
0.0
**
***
SKOV3-IP
HEY-A8
S3B
SKOV3-IP-NC
SKOV3-IP-shZEB2
HEY-A8-NC
HEY-A8-shZEB2
ZEB2
GAPDH
S3C
SKOV3-IP-NC
SKOV3-IP-shZEB2
HEY-A8-NC
HEY-A8-shZEB2
Relative mRNA levels
1.5
1.0
0.5
0.0
**
**
*
***
Nanog
Oct4
S3D
SKOV3-IP-NC
SKOV3-IP-shZEB2
HEY-A8-NC
HEY-A8-shZEB2
N-cad
VIM
GAPDH
S3E
HEY-A8
CD133⁻-NC
CD133⁺-NC
CD133⁻-shZEB2
CD133⁺-shZEB2
3.08%
7.19%
1.51%
3.68%
5.45%
9.39%
10.4%
4.01%
7-AAD
Annexin V
CD133⁻-NC
CD133⁻-shZEB2
CD133⁺-NC
CD133⁺-shZEB2
Percentage of apoptotic cells(%)
50
40
30
20
10
0
***
**
**
HEY-A8

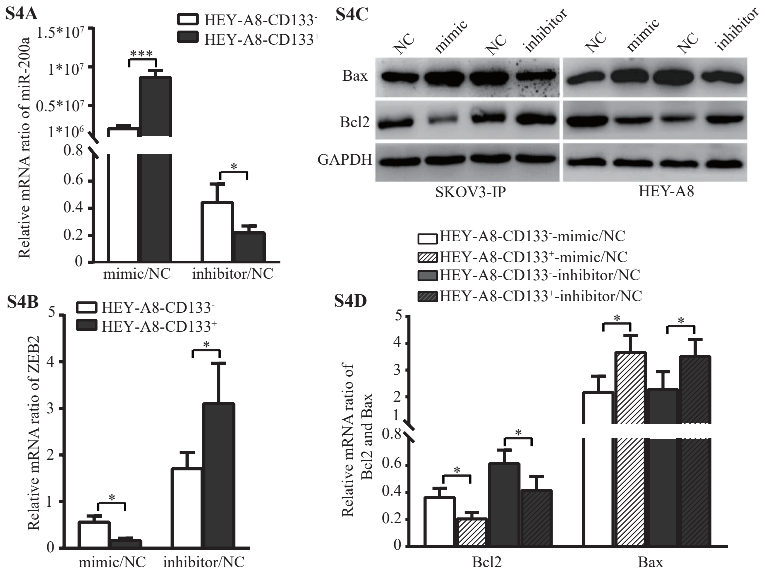
S4A
HEY-A8-CD133-
HEY-A8-CD133+
Relative mRNA ratio of miR-200a
1.5*10^7
1*10^7
0.5*10^7
1*10^6
0.8
0.6
0.4
0.2
0
***
*
mimic/NC
inhibitor/NC
S4B
HEY-A8-CD133-
HEY-A8-CD133+
Relative mRNA ratio of ZEB2
5
4
3
2
1
0
*
*
mimic/NC
inhibitor/NC
S4C
NC
mimic
NC
inhibitor
NC
mimic
NC
inhibitor
Bax
Bcl2
GAPDH
SKOV3-IP
HEY-A8
S4D
HEY-A8-CD133--mimic/NC
HEY-A8-CD133+-mimic/NC
HEY-A8-CD133--inhibitor/NC
HEY-A8-CD133+-inhibitor/NC
Relative mRNA ratio of Bcl2 and Bax
5
4
3
2
1
0.8
0.6
0.4
0.2
0
*
*
*
*
Bcl2
Bax
